# Granulocyte Colony-Stimulating Factor-Primed Unmanipulated Haploidentical Blood and Marrow Transplantation

**DOI:** 10.3389/fimmu.2019.02516

**Published:** 2019-11-01

**Authors:** Ying-Jun Chang, Xiang-Yu Zhao, Xiao-Jun Huang

**Affiliations:** ^1^Beijing Key Laboratory of Hematopoietic Stem Cell Transplantation, National Clinical Research Center for Hematologic Disease, Peking University Institute of Hematology, Peking University People's Hospital, Beijing, China; ^2^Peking-Tsinghua Center for Life Sciences, Beijing, China

**Keywords:** haploidentical stem cell transplantation, granulocyte colony-stimulating factor, poor graft function, relapse, virus infections

## Abstract

Granulocyte colony-stimulating factor (G-CSF), a growth factor for neutrophils, has been successfully used for stem cell mobilization and T cell immune tolerance induction. The establishment of G-CSF-primed unmanipulated haploidentical blood and marrow transplantation (The Beijing Protocol) has achieved outcomes for the treatment of acute leukemia, myelodysplastic syndrome, and severe aplastic anemia with haploidentical allografts comparable to those of human leukocyte antigen (HLA)-matched sibling donor transplantation. Currently, G-CSF-mobilized bone marrow and/or peripheral blood stem cell sources have been widely used in unmanipulated haploidentical transplant settings. In this review, we summarize the roles of G-CSF in inducing T cell immune tolerance. We discuss the recent advances in the Beijing Protocol, mainly focusing on strategies that have been used to improve transplant outcomes in cases of poor graft function, virus infections, and relapse. The application of G-CSF-primed allografts in other haploidentical modalities is also discussed.

## Introduction

**Figure 1:**
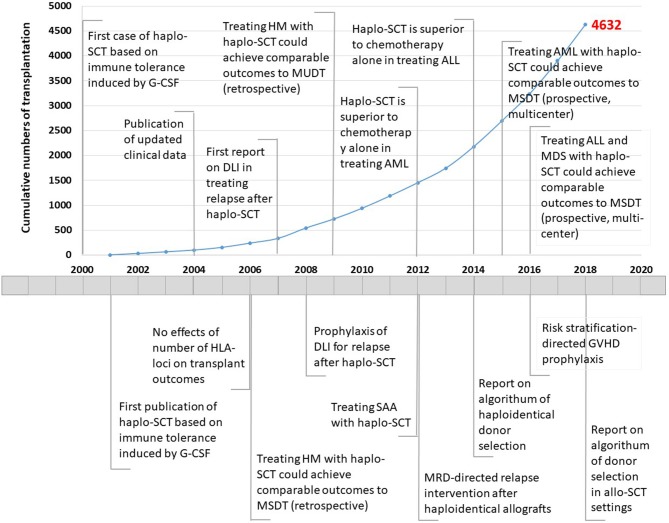
Timeline showing the number of haploidentical stem cell transplantation and advances in Peking University Institute of Hematology, 2000–2018. Haplo-SCT, haploidentical stem cell transplantation; G-CSF, granulocyte colony-stimulating factor; HLA, human leukocyte antigen; HM, hematological malignancies; MSDT, HLA-matched sibling donor transplantation; DLI, donor lymphocyte infusion; MUDT, HLA-matched unrelated donor transplantation; SAA, severe aplastic anemia; AML, acute myeloid leukemia; MRD, minimal residual disease; ALL, acute lymphoblastic leukemia; GVHD, graft-vs.-host disease. The red number indicates cumulative cases of patients who underwent haplo-SCT until December 31, 2018.

**Figure 2:**
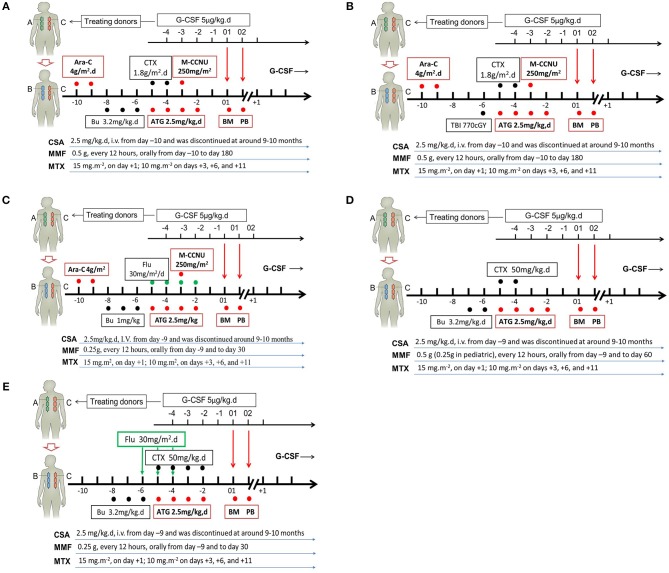
Individual conditioning regimens in the Beijing Protocol. Conditioning regimens for hematological malignancies without total body irradiation (TBI) **(A)**, or with TBI **(B)**, or with reduced intensity **(C)**; for severe aplastic anemia **(D)**; as well as for pediatric adrenoleukodystrophy and mucopolysaccharidosis **(E)**. G-CSF, granulocyte colony-stimulating factor; Ara-C, cytrarabine; CTX, cyclophosphamide; M-CCNU, Semustine; Bu, busulfan; ATG, thymoglobulin; BM, G-CSF-primed bone marrow harvests; PB, G-CSF-mobilized peripheral blood harvests; CSA, cyclosporine; MMF, mycophenolate mofetil; MTX, methotrexate; Flu, fludarabine.

Allogeneic stem cell transplantation (allo-SCT) is a method for the therapeutic cure of hematological malignancies (1–3). However, donor limitations restrict the wide use of allo-SCT. In the past two decades, researchers have established several haploidentical SCT (haplo-SCT) protocols based on different approaches to induce immune tolerance (4–6). Those approaches include *ex vivo* graft T cell depletion (TCD) in combination with megadoses of CD34^+^ cells and/or anti-third-party CD8 T cells, *in vitro* CD3αβ/CD19 depletion, immune tolerance induced by granulocyte colony-stimulating factor (G-CSF) (7), and post-transplantation cyclophosphamide (PT/CY) for tolerance induction (8–18). Based on T cell tolerance induced by G-CSF, the Peking University group established a novel G-CSF-primed haploidentical blood and marrow transplantation system (The Beijing Protocol, Figures 1, 2) (4, 5), including individualized conditioning regimens, the combination of unmanipulated G-CSF primed blood and marrow as allografts, donor selection based on non-human leukocyte antigen (HLA) systems, risk-directed strategies for graft-vs.-host disease (GVHD) (19), and relapse. Currently, because of the shift from TCD grafts to unmanipulated marrow and/or peripheral blood harvests, haplo-SCT is easier to

perform than before. The development and success of haploidentical allografts worldwide makes “everyone has a donor” a reality (20). Several reviews have already been published on this topic (21–25). Here, we summarize the advances in inducing T cell tolerance by treating healthy donors with G-CSF. We discuss the recent advances in the Beijing Protocol mainly focusing on strategies that have been used for poor graft function (PGF) (26–30), virus infections (31–33), and relapse (34–36). We also indicate the application of G-CSF-primed allografts for other haploidentical allograft modalities.

## T Cell Tolerance Induced by G-CSF

**Figure 3:**
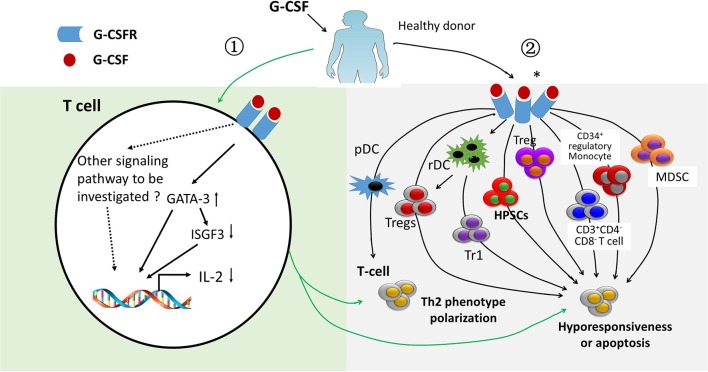
Immune regulatory effects of treating healthy donors with granulocyte colony-stimulating factor. Granulocyte colony-stimulating factor (G-CSF) has immune regulatory effects on T cells via direct (*light green area*) and indirect mechanisms (*light gray area*). ① ISGF-3 was down-regulated and GATA3 was up-regulated by G-CSF through binding G-CSF receptor, leading to a polarization of T cells from Th1 to Th2 phenotype as well as hyporesponsiveness of T cells (*light green area*). ② Effects of G-CSF obtained from G-CSF-primed allografts on polarizing T cell from Th1 to Th2. Suppressing T cell proliferation ability by regulatory T cells (Treg), type 1 Treg, CD34^+^ monocyte, myeloid derived suppressor cells, and CD3^+^CD4^−^CD8^−^ T cells obtained from G-CSF-primed allografts either via direct contact, cytokines, such as interleukin-10 and transform growth factor-β, or via other molecules, such as arginase-1 and reactive oxygen species (*light gray area*). *indicates G-CSF receptor expressed on immune cells, such as myeloid-derived suppressor cells.

G-CSF has been widely applied to mobilize hematopoietic stem/progenitor cells in allo-HSCT settings. In the past 20 years, a number of studies support the notion that G-CSF plays an important role in regulating immune cell number and function in allografts, especially in inducing T cell tolerance (37–46) Previously, researchers mainly focused on the regulatory effects of G-CSF on T cells through indirect effects, such as expanding regulatory T cells, CD34^+^ regulatory monocytes, tolerogeneic antigen presentation cells, regulatory B cells (47), CD3^+^CD4^−^CD8^−^ T cells, regulatory γδ T cells (48), suppressor interleukin-10 (IL-10)^+^ neutrophils, myeloid-derived suppressor cells (MDSCs) (37), and granulocytic MDSCs (also known as low-density neutrophils) (49). All of these cells could suppress T cell proliferation through IL-10, transforming growth factor-β (TGF-β), nitric oxide (NO), indoleamine 2,3-dioxygenase (IDO), and/or cell contact (Figure 3).

In 2003, Franzke et al. (45) suggested that G-CSF acts as a strong immune regulator in T cells and directly modulates T-cell immune responses via its receptor on T cells. They demonstrated that G-CSF could limit the interferon-γ signaling in T cells by suppressing the gene expression of ISGF3-γ subunit/p48 in CD4^+^ donor T cells. *In vitro* experiments also showed that the Th2 type could be induced by G-CSF through direct induction of GATA-3. In 2014, *in vivo* experiments also demonstrated that donor T cell alloreactivity could be modulated directly via binding to G-CSF receptor expressed on T cells (43). The authors reported that the protective effects of G-CSF on GVHD imparted during stem cell mobilization were totally dependent on direct signaling through the T cell, because WT but not G-CSFR^−/−^ donor T cells were modulated by G-CSF (43). Overall, mounting evidence indicates that G-CSF can induce T cell tolerance through both indirect and direct pathways (Figure 3) (43, 45, 46), although the detailed molecular mechanisms of which remain unclear.

Clinically, Ringdén et al. (50) reported that application G-CSF after bone marrow transplantation increased the cumulative incidence of grades II to IV acute GVHD, which is not consistent with above-mentioned concept. The following reasons may account for this inconsistence: First, use of G-CSF after transplantation increases the levels of soluble interleukin-2 (IL-2)-receptor-alpha that may aggravate acute GVHD (51). Second, treating healthy donors with G-CSF decreases the production of tumor necrosis factor alpha, IL-2, and interferon-γ; the immunoregulatory effects of G-CSF on cytokines, T cells, and regulatory cells of donors might contribute to the lower incidence of GVHD after using G-CSF-primed bone marrow harvests and/or G-CSF-mobilized peripheral blood harvests as allografts (39, 46).

## Recent Advances in the Beijing Protocol

The Beijing Protocol, one of the main haplo-SCT modalities, has been performed in nearly 98% of transplant centers in China (52). In the first half of 2016, the proportion of haplo-SCT out of the allo-HSCT had increased to 51.7% according to data reported by Xu et al. (52) on behalf of the Chinese Blood and Marrow Transplantation Register Group. Based on the Beijing experience, Di Bartolomeo et al. (53) treated 80 patient hematological malignancies by applying a modified protocol. The key characteristics of their protocol were (i) a chemotherapy-based conditioning regimen, (ii) infusion of only G-CSF-stimulated bone marrow harvests, (iii) intensified GVHD prophylaxis with anti–thymocyte globulin (ATG), cyclosporine (CSA), methotrexate (MTX), mycophenolate mofetil (MMF), and anti-CD25 antibody. Di Bartolomeo et al. (53) found that the cumulative incidence (CI) of neutrophil engraftment was 93%. The 100-day CI for II–IV grade of acute GVHD was 24%. The 2-year CI of extensive chronic GVHD was 6%. The 3-year probability of overall survival (OS) and disease-free survival (DFS) for standard-risk and high-risk patients was 54 and 33% and 44 and 30%, respectively.

In a multicenter, prospective study, Wang et al. (54) showed that unmanipulated haplo-SCT achieved outcomes, including 3-year DFS (74 vs. 78%, *P* = 0.34) and OS (79 vs. 82%, *P* = 0.36), similar to those of HLA-matched sibling donor transplantation (MSDT) for acute myeloid leukemia patients in complete remission 1 (CR1). In another biologically phase III randomized study, Wang et al. (55) demonstrated that the 3-year DFS (61 vs. 60%, *P* = 0.91) and OS (68% vs. 66%, *P* = 0.81) were comparable between adults with Philadelphia-negative high-risk acute lymphoblastic leukemia (ALL) who underwent haplo-SCT and those who received MSDT. Similar to the report by Wang et al. (55), adults with standard-risk ALL in CR1 who underwent haplo-SCT could also achieve comparable survival to those of MSDT (56). In a registry-based study, Wang et al. (57) reported that the 4-year adjusted probabilities of OS (63 vs. 73%) and relapse-free-survival (63% vs. 71%) between patients who received haplo-SCT with 4–5/6 matched donors and those who received MSDT were comparable. These results were also confirmed by researchers from other centers in China (58). In Europe and the United States of America, a number of studies obtained similar results as reported by us (54, 55, 57), that patients with hematological malignancies who underwent haplo-SCT with PT/CY could achieve comparable outcomes to those with MSDT or HLA-matched unrelated donor transplantation (59–65).

In the past 3 years, a series of studies from different transplant centers in China confirmed the efficacy, safety, and feasibility of treating adult and pediatric severe aplastic anemia (SAA) patients with haploidentical allografts based on G-CSF-induced immune tolerance (Table 1) (66–75). In a multicenter prospective study, Xu et al. (76) showed that treating SAA patients (*n* = 101) with haplo-SCT could achieve a 3-year estimated OS (89.0 vs. 91.0%, *P* = 0.555) and failure-free survival (FFS, 86.8 vs. 80.3%, *P* = 0.659) when compared with patients (*n* = 48) who received contemporaneous transplantation from matched related donors. In another registry-based study, Xu et al. (67) further observed similar 3-year estimated OS (86.1 vs. 91.3%, *P* = 0.358) and FFS (85.0 vs. 89.8%, *P* = 0.413) between the haplo-SCT and MUDT cohorts for the treatment of SAA. In this regard, Professor Neal S. Young commented that “*Haploidentical transplantation has been advocated in China as first-line treatment for children*” (77).

**Table 1:** Recent^*^ trials and results of non-hematological malignancies treated with haploidentical allografts based on immune tolerance induced by G-CSF.

**References**	**Pts (No.)**	**Diagnosis**	**Graft**	**ANC**	**PLT**	**GVHD**		**TRM**	**Relapse**	**FFS**	**OS**
						**Acute II–IV**	**cGVHD**				
Xu et al. (68)	52	SAA	G-BM+G-PB	13 (10–21)	14 (7–180)	39.2%	38.1%	15.5% at 1 yr	NA	82.7 at 3 yr	84.5 at 3 yr
Xu et al. (67)	89	SAA	G-PB+G-BM (87.6%)	12 (9–20)	15 (6–91)	30.34%%	39.3% at 3 yr	NA	NA	85.0% at 3 yr	86.1% at 3 yr
Liu et al. (66)	44	SAA	G-BM+G-PB	12 (8–21)	19 (8–154)	29.3%	17.1%	NA	NA	NA	77.3% at 2 yr
Zeng et al. (75)	115	SAA	G-BM+G-PB	13 (9–25)	14 (8–82)	34.5%	18.5% at 5 yr	NA	NA	NA	74.8% at 5 yr
Sun et al. (78)	8	Thalassaemia major	G-PB	10 (10–15)	13 (10–102)	3/8	1/8	0/8	NA	100% at 1 yr	100% at 1 yr
Li et al. (65)	34	SAA	G-BM+G-PB (94.1%)	13 (10–20)	16.5 (7–30)	14.8%^#^	26.47%	NA	NA	93.3% at 5 yr	79.4% at 5 yr
Li et al. (69)	119	SAA	G-PB+G-BM (73%)	12 (8–22)	14 (9–154)	30%	27%	NA	NA	NA	75% at 3 yr
Lu et al. (70)	41	SAA	G-PB+G-BM	14 (10–21)	13 (3–56)	27%	39%	NA	NA	76.4% at 3 yr	80.3% at 3 yr
Chen et al. (79)	6	IMDs	G-PB+G-BM	12 (11–13)	12 (10–15)	4/6	1/6	0/6	NA	NA	100% at 1 yr
Wang et al. (72)	35	SAA	G-PB	14 (10–22)	18 (9–36)	25.71%	38.58%	14.29%	NA	NA	85.71% at 2 yr
Yang et al. (74)	20	SAA	G-PB+G-BM (50%)	16 (11–26)	19 (10–34)	40%	15%	NA	NA	80% at 3 yr	85% at 3 yr

* *Published between 2017 and 2019. G-CSF, granulocyte colony-stimulating factor; Pts, patients; No., number; ANC, absolute neutrophil count; PLT, platelet; GVHD, graft-vs.-host disease; cGVHD, chronic GVHD; TRM, transplant-related mortality; FFS, failure-free survival; OS, overall survival; SAA, severe aplastic anemia; G-BM, granulocyte colony-stimulating factor (G-CSF)-primed bone marrow; G-PB, G-CSF-mobilized peripheral blood stem cell grafts; NA, not available; yr, year; IMDs, inherited metabolic storage diseases*.

# *indicate grades III–IV acute GVHD*.

More recently, the G-CSF-based haplo-SCT protocol has been successfully used for the treatment of inherited metabolic storage diseases (IMD) and genetic diseases, such as adrenoleukodystrophy (ALD), mucopolysaccharidosis (MPS), and thalassaemia major (78, 79). In China, six IMD cases, including ALD (*n* = 4) and MPS (*n* = 2), were treated by Chen et al. (79) with busulfan (Bu), fludarabine (Flu), and cyclophosphamide (Cy) conditioning regimen. Hematopoietic reconstitution was achieved in all cases. Four patients developed grade I–II acute GVHD, and one patient had limited chronic GVHD. After a median follow-up of 292 days, the cumulative incidence of 1-year transplant-related mortality (TRM) was 0%. The 1-year probability of OS was 100%. In another study, Sun et al. (78) reported the results of eight thalassaemia major children who underwent haplo-SCT using the FBCA conditioning regimen [Flu, Bu, Cy, and antithymocyte globulin (ATG)]. All cases achieved hematopoietic recovery after transplantation. Four (50%) and two (25%) cases experienced grade I–II and grade III–IV acute GVHD, respectively. One patient suffered from localized chronic GVHD of the skin. All cases survived and achieved independence from blood transfusion. The OS and transfusion-free survival rates were both 100% after a median follow-up of 36 months. These studies suggest that haplo-SCT based on G-CSF could be a feasible, safe, and efficient approach for the treatment of IMD and genetic disease.

Although the indications for haplo-SCT have been extended, and no differences in the outcomes between haploidentical allografts, that is, MSDT and MUDT, were achieved, transplant complications, such as PGF (4, 26–30, 80–83) virus infection (31, 32, 84), and relapse (85–94), remain the major causes of morbidity and mortality. Fortunately, in recent years, some advances in the treatment of PGF, virus infections, and leukemia relapse after haplo-SCT have been made by scholars worldwide. We discuss these advances here.

### Mechanisms and Therapies for PGF

PGF, which occurs in 5–26% of cases after allo-HSCT (26, 28–30) has become a growing obstacle that contributes to high morbidity and mortality. PGF is defined as follows: absolute neutrophil count ≤0.5 × 10^9^/L, platelets ≤20 × 10^9^/L, or hemoglobin ≤70 g/L for at least three consecutive days beyond day 28 post-transplantation with a transfusion requirement associated with hypoplastic–aplastic BM in the presence of complete donor chimerism. Previous studies by Ciurea et al. (85) showed that patients with high donor-specific anti-HLA antibody (DSA) levels (>5,000 MFI) and complement-binding DSA antibodies (C1q positive) experienced higher risk of primary graft failure. Our data demonstrated that a number of risk factors, such as infused CD34^+^ cells, DSA, GVHD, and CMV infection, were associated with PGF (86, 87). The available therapeutic strategies for PGF patients include the administration of hematopoietic growth factors, donor lymphocyte infusion (30), a second allo-HSCT, a CD34^+^ cell boost (29, 30), or mesenchymal stem cell (MSC) infusion (80, 88). Additionally, eltrombopag, an oral thrombopoietin receptor agonist, has shown promising results in severe aplasia anemia. In a recent study, 12 patients who responded poorly to standard treatments for secondary PGF after allo-SHCT were treated with eltrombopag (89). The median duration from PGF diagnosis to eltrombopag treatment was 59 (range, 30–180) days. The dose of eltrombopag was 25 mg/day for 3 days and subsequently increased to 50 or 75 mg/day. After treatment for 8 (range, 2–23) weeks, 10 cases responded to eltrombopag: eight cases achieved complete response (CR) and two cases achieved partial response. The median time from treatment to achieving CR was 29 (10–49) days. The 1-year probability of OS was 83.3%. No TRM and no evidence of cataract, thrombosis, or any other grade 3/4 toxicities were observed (89). This result was also confirmed by Fu et al. (90) and Marotta et al. (91), suggesting that eltrombopag could be an alternative treatment for PGF.

Several studies have been reported seeking to elucidate the pathophysiology of PGF (4, 26, 27, 81–83). First, patients with PGF after allo-SCT had reduced and dysfunctional endothelial progenitor cells (EPCs) in the bone marrow microenvironment: these EPCs are characterized by impaired proliferation, migration, angiogenesis, and increased levels of ROS and apoptosis. In addition, the increased reactive oxygen species (ROS) could activate p38 and its downstream transcription factor in BM EPCs, both of which might contribute to the occurrence of PGF. Second, BM CD34^+^ cells are functionally normal in PGF; however, elevated ROS in CD34^+^ cells might lead to exhaustion of quiescent BM CD34^+^ cells. Third, dysregulated T cell responses, including a shift in the Th1/Th2 and Tc1/Tc2 ratios toward a type 1 response and an increased Th17/Treg ratio, may also be involved in the pathogenesis of PGF. In addition, the presence of DSA may contribute to primary PGF through antibody-dependent cell-mediated cytotoxicity, resulting in impairment or apoptosis of CD34^+^ cells in patients with PGF (92).

According to previous studies (4, 26, 27, 81–83). Shi et al. (26) and Wang et al. (27) found that atorvastatin, a regulator of p38 MAPK, may offer a novel therapeutic strategy to promote hematopoietic recovery through repair of the BM microenvironment in PGF patients. More recently, our group performed two prospective clinical trials. In the first one (*n* = 68), Kong et al. (4) found that EC < 0.1% in the BM before transplantation identified high-risk patients with PGF and PT. In the second one (*n* = 35), cases with EC < 0.1% were treated with oral *N*-acetyl-l-cysteine (NAC; 400 mg, three times per day) from −14 days to +60 days continuously (experiment group); the remaining cases with EC ≥ 0.1% (*n* = 39) underwent allo-HSCT only (control group). The authors observed a similar survival rate at 10 months after transplantation between the experiment and control groups (4). This study suggests that improvement of the BM microenvironment through EC-directed NAC intervention could be a promising approach to enhance hematopoietic recovery in allo-HSCT settings. Therefore, a randomized, controlled, multicenter study is warranted.

### Prophylaxis and Treatment of Virus Infections

In haplo-SCT with G-CSF modality, the cumulative incidence of cytomegalovirus (CMV) DNAemia varies from 63.7 to 66.1%, which remains one of the main causes of morbidity and mortality. The risk factors for CMV DNAemia include HBsAg seropositivity, acute GVHD before CMV DNAemia, and poor CMV-specific CD8^+^ T central memory subset recovery (93). In contrast, transplantation from HLA-mismatched family donors (*P* < 0.001), acute GVHD (*P* < 0.001), and donor-recipient KIR ligand mismatched (*P* = 0.012) were associated with an increased risk of refractory CMV infection (31, 32). In addition, refractory CMV infection within 60–100 days after allo-HSCT was an independent risk factor for NRM (*P* = 0.015). Compared to placebo, letermovir prophylaxis can significantly reduce the incidence of CMV disease. For cases with refractory CMV infection/reactivation or who failed ganciclovir, foscarnet, and cidofovir, these adoptive T-cell therapies, for example, CMV-specific T-cell (CMV CTL), represent a promising approach.

In a recent study, 32 patients with refractory CMV infection following haplo-SCT were treated with adoptive transfer of CMV CTL. Pei et al. (33) showed that 27 of the 32 cases exhibited CMV clearance within 4 weeks after treatment without recurrence. Compared with those of the non-refractory CMV-infected patients, the authors observed significantly fewer CMV-specific CD8^+^IFN-γ^+^ and CD4^+^IFN-γ^+^ T cells. In addition, the CMV clearance is closely correlated with rapid and massive expansion of CD8^+^ and CD4^+^ CMV CTL *in vivo*. Using a humanized HCMV-infected mouse model, the same group further elucidated that systemic HCMV infection could be combated after first-line therapy with CTL through *in vivo* promotion of the recovery of graft-derived endogenous HCMV-specific CTL (84). These studies provide substantial evidence suggesting that CMV infection could be successfully addressed with prophylaxis, treatment, and adoptive transfer of CMV CTL (84). In summary, future studies should focus on the risk factor-directed intervention or development of new drugs for CMV infections in haplo-SCT settings.

### Minimal Residual Disease (MRD)-Based Transplant Indication to Decrease Relapse

MRD determined by multiparameter flow cytometry (MFC) and/or real-time polymerase chain reaction (RT-PCR) at pre- and post-transplantation could be used for predicting outcomes (Table 2) (5, 94–105) Our group showed that, after two course consolidation therapies, patients with *t*_(8;21)_ AML could be classified as the low-risk group or high-risk group (106). The low-risk group was defined as cases who achieved major molecular remission (MMR)/MRD negativity [>3-log reduction in RUNX1/RUNX1T1 transcripts (<0.4%) compared with the pre-treatment baseline of 388% in our center] after the second consolidation therapy and maintained MMR for 6 months thereafter. The high-risk group was defined as cases not achieving MMR/MRD positivity after the second consolidation therapy or those exhibiting the loss of MMR (defined as RUNX1/RUNX1T1 transcript levels >0.4% in MMR patients) within 6 months of achieving MMR. In the high-risk subgroup, compared with cases receiving chemotherapy alone, cases who underwent allo-HSCT experienced a significantly lower cumulative incidence of relapse (CIR, 22.1 vs. 78.9%, *P* < 0.0001) and superior DFS (61.7 vs. 19.6%, *P* = 0.001). However, allo-HSCT was not superior to chemotherapy alone in the low-risk group. Multivariate analysis demonstrated that MRD status and treatment strategy were independent risk factors for CIR and DFS (106). Our results suggest that MRD-directed risk stratification treatment may improve the outcome not only of patients with AML with *t*_(8;21)_ in CR1 but also of *t*_(8;21)_ AML cases after two courses of consolidation therapy; thus, allo-HSCT should be performed for those with positive MRD.

**Table 2:** Correlation of MRD with clinical outcomes in patients who underwent Haplo-SCT.

**References**	**Diagnosis (Pt. No.)**	**Transplant modalities**	**Methods for MRD**	**Transplant outcomes**	**Multivariate analysis**
Zhao et al. (94)	ALL (543)	Haplo-SCT based on G-CSF	MFC	Positive pre-MRD, except for low level one (MRD < 0.01%), is correlated with higher CIR, and inferior LFS.	Yes
Lv et al. (5)	Intermediate risk AML (78)	Haplo-SCT based on G-CSF	MFC	Positive MRD (detectable) after two-cycle consolidation is associated with higher CIR and inferior survival.	Yes
Liu et al. (95)	AML (460)	Haplo-SCT based on G-CSF	MFC	Peri-transplantation MRD (detectable) assessment is useful for risk stratification.	Yes
Liu et al. (96)	AML (145)	Haplo-SCT based on G-CSF	MFC	Persistent positive MRD (detectable) pre-transplantation predicts poor clinical outcome.	Yes
Canaani et al. (97)	AML (393)	Haplo-SCT based on G-CSF (27.2%) Haplo-SCT with PTCy (66%) Haplo-SCT with G-CSF+PTCy (6.8%)	MFC	Positive pre-transplant MRD status (detectable) is a predictor of poor prognosis.	Yes
Qin et al. (100)	AML (14)	Haplo-SCT based on G-CSF (79%) MSDT (21%)	RT-PCR	TLS-ERG transcript levels (>1.0%) predict high-risk of relapse and inferior survival.	No
Hong et al. (104)	B-ALL (28)	Haplo-SCT based on G-CSF (90%) MSDT (10%)	TR-PCR	The E2A-PBX1 positive (detectable) after transplantation is correlated with poor prognosis.	No
Tang et al. (103)	AML (53)	Haplo-SCT based on G-CSF (75.5%) MSDT (24.5%)	RT-PCR	Post-transplant CBFB-MYH11 positive (defined as ≤ 3-log reduction in CBFB-MYH11 transcripts compared with the pre-treatment baseline level) could predict poor outcomes.	No
Zhao et al. (101)	T-ALL (29)	Haplo-SCT based on G-CSF (90%) MSDT (10%)	RT-PCR	Pre- or post-transplantation SIL-TAL1 positive (detectable) is associated with higher CIR and inferior DFS and OS.	No
Liu et al. (105)	AML (16)/ALL (24)	Haplo-SCT based on G-CSF (75%) MSDT (10%) Other alternative modality (15%)	RT-PCR	MLL gene positive after transplantation (detectable) is associated with higher CIR and inferior DFS and OS.	Yes
Wang et al. (102)	ALL (92)	Haplo-SCT based on G-CSF (48%) MSDT (48%) Other alternative modality (4%)	RT-PCR	Positive MRD (defined as ≤ 3-log reduction in RUNX1/RUNX1T1 transcripts when compared with the pre-treatment baseline level) at 1, 2, and 3 months after transplantation predicts higher CIR and inferior survival.	Yes
Zhou et al. (99)	ALL (139)	Haplo-SCT based on G-CSF (76%)MSDT (24%)	MFC	Positive MRD post-transplantation (detectable) is associated with high risk of relapse and inferior survival.	Yes
Zhou et al. (98)	AL (138)	Haplo-SCT based on G-CSF (58%) MSDT (29%) Other alternative modalities (13%)	RT-PCR	The WT1 expression level (≥0.60%) after transplantation is associated with higher CIR and inferior survival.	Yes

*MRD, minimal residual disease; Haplo-SCT, haploidentical stem cell transplantation; Ref., reference; Pt., patients; No., number; G-CSF, granulocyte colony-stimulating factor; MFC, multiparameter flow cytometry; CIR, cumulative incidence of relapse; LFS, leukemia-free survival; AML, acute myeloid leukemia; PTCy, post-cyclophosphamide; RT-PCR, real-time quantitative polymerase chain reaction; MDST, human leukocyte antigen-matched sibling donor transplantation; AL, acute leukemia*.

More recently, another multicenter study enrolled 229 AML patients with NPM1-mutated (NPM1m). Balsat et al. (107) reported that a >4-log reduction in PB-MRD was significantly associated with a higher relapse incidence and shorter OS. The DFS and OS were significantly improved by allo-HSCT in those with a >4-log reduction in PB-MRD. These data suggest that NPM1m PB-MRD may be used as a predictive factor for allo-HSCT indication. Considering the comparable outcomes between haplo-SCT and MSDT, the data reported by Balsat et al. (107) and us (106) suggest that for the abovementioned MRD-positive AML patients who lack MSD, haplo-SCT might be an alternative choice to improve clinical outcomes if there are no MSDs available.

### MRD-Based Donor Selection to Decrease Relapse

In the era of haplo-SCT, MSDT remains the first choice for transplant candidates according to recent literatures (23, 108). Recently, Chang et al. (109) showed that, for AML patients with positive pre-transplantation (pre-MRD), haplo-SCT could achieve lower CIR (19 vs. 57%, *P* < 0.001) and superior LFS (73 vs. 29%, *P* < 0.001) compared with those after MSDT in the retrospective group and prospective group (CIR, 13 vs. 36%, *P* = 0.017 and LFS, 80 vs. 48%, *P* = 0.007, respectively). These results were further confirmed in pediatric patients (110) and a subset of AML cases with FLT3-ITD (111), indicating superior graft-vs.-leukemia (GVL) effects of haploidentical donors to HLA-matched sibling donors. In another study, 64 Hodgkin lymphoma (HL) patients who relapsed following autologous SCT were treated with haplo-SCT with PT-Cy (*n* = 30) and MSDT (*n* = 34). After a median follow-up of 47 months, Mariotti et al. (112) found that patients receiving haplo-SCT experienced lower 3-year CIR (13 vs. 62%, *P* = 0.0001) and better PFS (60 vs. 29%, *P* = 0.04). The authors also indicated that haplo-SCT (HR, 0.17, *P* = 0.02) was independently associated with a reduced risk of relapse. In a more recent study, 151 consecutive cases with HL who were treated with haplo-SCT (*n* = 61) or MSDT (*n* = 90) were retrospectively enrolled. Gauthier et al. (113) reported a significantly lower GVHD-free/relapse-free survival (GRFS) in the MRDT group compared with those of haplo-SCT based on the PT/CY group (HR = 0.339, *P* < 0.001).

In contrast to the studies reported by other researchers and us, Ringden et al. (114) observed similar risk of relapse between acute leukemia patients who received haploidentical donor grafts (*n* = 864) and those given MSD transplants (*n* = 9,815), suggesting a similar GVL effect. The different results reported by Ringdén et al. and us may be related to the differences in patient population, conditioning regimen, allografts infused, and GVHD prophylaxis.

In summary, studies by others (36, 112, 113, 115) and us (109, 111) have suggested the inferiority of MSDT to haplo-SCT, indicating that, for AML patients with positive pre-MRD and HL, haploidentical donors might be selected first in experienced centers, although controversy remains. Therefore, a prospective, randomized study is needed to elucidate which one has better anti-leukemia activity, MSDT or haplo-SCT?

### MRD-Directed DLI to Decrease Relapse

The transplant outcomes are worse for patients who had a hematological relapse after allo-HSCT, including haplo-SCT (116–119). In the past 10 years, a modified DLI (mDLI) protocol was used to treat patients with G-CSF-mobilized peripheral blood harvests, followed by short-term immune suppression, including cyclosporine (CSA) or methotrexate (MTX) (34, 35). Compared with traditional DLI, mDLI alleviated the pancytopenia and reduced acute GVHD without influencing the GVL effects. The safety and efficacy of mDLI for prophylaxis and treatment of relapse after haplo-SCT have been well-established. In a prospective study, 814 patients with standard-risk acute leukemia were enrolled. Yan et al. (34) reported that the MRD-positive patients who had mDLI had comparable 3-year CIR (27.8 vs. 18.1%) and DFS (55.6 vs. 61.6%) compared with those MRD-negative patients. The authors found that factors associated with lower CIR included MRD negative after transplantation (OR = 0.255, *P* < 0.001) and receiving DLI (OR = 0.269, *P* < 0.001). Factors correlated with superior DFS included receiving DLI (OR = 0.436, *P* = 0.006) and MRD negative after transplantation (OR = 0.511, *P* = 0.001). In a recent study, Yan et al. (35) further showed that, for subjects with refractory/relapsed acute leukemia, MRD- and GVHD-guided multiple DLIs could reduce CIR and improve LFS and OS.

Presently, a number of strategies, including DLI, cellular approaches (NK cells and CAR-T) (117), targeted drugs, hypomethylating agents, IFN-γ, and blinatumomab (120–122), are currently applied for relapse prevention or treatment in the clinic (116–119). Blinatumomab, a CD3 × CD19 bispecific antibody, has been approved previously for the treatment of relapsed or refractory B-cell precursor ALL (BCP-ALL). In a recent study (*n* = 116), adults with BCP-ALL in hematologic CR with MRD (≥10^−3^) received blinatumomab 15 μg/m^2^/day by continuous IV infusion for up to four cycles. Patients could undergo allo-HSCT at any time after cycle 1. Gökbuget et al. (122) found that 88 (78%) of 113 evaluable patients achieved a complete MRD response. The RFS at 18 months was 54%. Grade 3 or 4 neurologic events occurred in 10 and 3% of cases, respectively. Four patients (3%) had cytokine release during cycle 1. These data suggest that blinatumomab could be used not only in treating relapse but only in intervention cases with positive MRD because those responders had significantly longer RFS and OS compared to non-responders. Overall, the available data (34, 35, 123) suggest that MRD-directed relapse intervention could be a simple method in haplo-SCT settings, leading to improved outcomes.

## Application of G-CSF-primed Allografts in Haplo-SCT With PTCy

For the unmanipulated haplo-SCT protocol with PTCy using steady-state bone marrow (SS-BM) as allografts (124), 68 patients with hematological malignancies were enrolled. The authors reported that the CIR at 1 and 2 years following transplantation was 51 and 58%, respectively. Therefore, a number of studies use G-CSF-mobilized peripheral blood harvests (G-PB) as allografts (Table 3) (7–17, 125). In a recent multicenter retrospective study from the United States, Bashey et al. (16) compared the outcomes of patients with hematological malignancies who underwent haplo-SCT based on PTCy receiving G-PB (*n* = 190) and SS-BM (*n* = 481). The authors found that there were no significant differences in NRM (G-PB 16% vs. SS-BM 17%, *P* = 0.78) and OS (G-PB 57% vs. SS-BM 54%, *P* = 0.52); however, compared to those with SS-BM, cases receiving G-PB experienced a significantly higher incidence of grades II–IV acute GVHD (G-PB 42% vs. SS-BM 25%, *P* < 0.001) and 2-year chronic GVHD (G-PB 41% vs. SS-BM 20%, *P* = 0.001). The authors demonstrated that patients receiving G-PB had a lower CIR (G-PB 28% vs. SS-BM 45%, *P* < 0.001) and superior progression-free survival (G-PB 54% vs. SS-BM 41%, *P* = 0.002).

**Table 3:** Informative trials and results regarding G-CSF-primed allografts used in haplo-SCT with PTCy.

**Reference**	**Pts (No.)**	**Diagnosis**	**Median (range) age, yr**	**Conditioning regimen**	**Graft**	**GVHD**		**TRM**	**Relapse**	**DFS**	**OS**
						**Acute II–IV**	**cGVHD**				
Solomon et al. (125)	20	HM	44 (25–56)	MAC	G-PB	30%	35%^*^	10% at 1 yr	40% at 1 yr	50% at 1 yr	69% at 1 yr
Raj et al. (9)	55	HM+SAA	49 (14–69)	RIC	G-PB	61% at 1 yr^#^	18% at 2 yr	23% at 2 yr	28% at 2 yr	66% at 1 yr	78% at 1 yr
Nakamae et al. (8)	20	HM	47 (18–65)	MAC	G-PB	60%	10%	11% at 1 yr	53% at 1 yr	35%^*^ at 1 yr^**†**^	55% at 1 yr
Sugita et al. (10)	31	HM	48 (21–65)	RIC	G-PB	23%	15% at 1 yr	23% at 1 yr	45% at 1 yr	34% at 1 yr	45% at 1 yr
Jaiswal et al. (11)	20	HM	12 (2–20)	MAC	G-PB	35%	5%	20% at 1 yr	25.7% ^**^	59.2% at 2 yr	64.3% at 2 yr
Moiseev et al. (12)	86	AML/ALL	34 (18–59)	MAC	G-PB	19%	16%	16% at 2 yr	19% at 2 yr	65% at 2 yr	69% at 2 yr
González-Llano et al. (13)	25	HM	10 (1–21)	MAC	G-PB	43%	15%	36% at 1 yr	40% at 1 yr	33% at 1 yr ^**†**^	50% at 1 yr
Bashey et al. (16)	190	HM	47 (19–73)	MAC	G-PB	42% at 6 mon	41% at 2 yr	17% at 2 yr	28% at 2 yr	54% at 2 yr	57% at 2 yr
Hong et al. (14)	34	HM	11.1 (0.9–20.3)	MAC	G-PB	38.2%	9.1% at 2 yr	2.9% at 2 yr	21.7% at 2 yr	79.4% at 2 yr	85% at 2 yr
Ruggeri et al. (15)	191	AML/ALL	18.3 (1.6–50.5)	RIC (51%)	G-PB	28%	35% at 1 yr	23% at 2 yr	22% at 2 yr	51% at 2 yr	55% at 2 yr
Granata et al. (16)	181	HM	60 (19–73)	MAC	G-PB	23%	17% at 2 yr	21% at 2 yr	17% at 2 yr	62% at 2 yr	66% at 2 yr
Sugita et al. (17)	127	HM+Others	36 (17–60)	MAC (39%)	G-PB	18%	36% at 2 yr	10% at 2 yr	36% at 2 yr	54% at 2 yr	68% at 2 yr
			58 (22–65)	RIC (61%)	G-PB	14%	27% at 2 yr	20% at 2 yr	45% at 2 yr	35% at 2 yr	44% at 2 yr

*Haplo-SCT, haploidentical stem cell transplantation; PTCy, post-cyclophosphamide; Pts, patients; No., number; GVHD, graft-vs.-host disease; cGVHD, chronic GVHD; TRM, transplant-related mortality; DFS, disease-free survival; OS, overall survival; HM, hematological malignancies; MAC, myeloablative conditioning regimen; G-PB, G-CSF-mobilized peripheral blood stem cell grafts; yr, year; SAA, severe aplastic anemia; RIC, reduced intensity conditioning regimen*.

* *indicates that the cumulative incidence of cGVHD was 35% after 20 months (range: 10–36 months) for surviving patients*.

† *indicates that the estimated 1-year EFS is 35 and 33%, respectively, for these two studies*.

# *indicates that the cumulative incidences of grade II and grade III acute GVHD at 1 year were 53 and 8%, respectively*.

** *indicates that at a median of 185 days, a cumulative incidence of disease progression was 25.7%*.

More recently, a meta-analysis of four comparative retrospective reports and 10 single-arm reports, with a total of 1,759 patients (462 patients received PBSCT, and 1,297 patients received BMT) who received haplo-SCT with PTCy, was performed by Yu et al. (126) They reported that compared with those of the BM group, patients in the PB group experienced a significantly higher incidence of grade II–IV (OR = 1.778) and grade III–IV acute GVHD (OR = 1.741), as well as rapid hematopoietic recovery (OR = 1.843). No significant differences in 2-year CIR, OS, DFS, and chronic GVHD between the two groups were observed. Considering comfort, safety, and speed, G-PB is suitable for haplo-SCT and is currently widely used in the settings of haploidentical allograft with PTCy (126). Thus, multicenter, prospective, randomized studies are warranted to evaluate whether G-PB or BM is the best allograft in the setting of haplo-SCT with PTCy.

## Future Direction

There are several questions that remain to be answered in the field of G-CSF-primed unmanipulated haploidentical blood and marrow transplantation. First, the mechanisms underlying T cell immune tolerance induced by G-CSF remain to be further investigated. Second, G-CSF-primed bone marrow harvests and/or peripheral blood stem cell harvests have been widely used in unmanipulated haploidentical allografts based on G-CSF or haplo-SCT with PTCy. However, we do not know which is the best stem cell source: G-CSF-primed bone marrow harvests, G-CSF-mobilized peripheral stem cell harvests, or a mixture of allografts of both of these harvests? Third, the contributions of DSA, that is, impairment of the cell immune and bone marrow niche, to PGF have been identified. More efforts are needed to investigate which methods, available drugs or novel ones, could prevent or treat PGF based on known immune mechanisms underlying this complication. Fourth, more clinical data, especially multicenter, prospective, randomized trials, are needed to confirm the results that haplo-SCT has a stronger GVL effect compared with MSDT. In addition, the immunological mechanism underlying relapse after transplantation and the stronger GVL effects of haplo-SCT remain to be elucidated. Finally, regarding the MRD-directed intervention, the perfection of MRD detection methods and establishment of novel intervention strategies, such as new generation CAR-T, will further improve transplant outcomes.

In summary, several haplo-SCT protocols have been established worldwide (Tables 1, 3), but each one has both disadvantages and advantages. No haplo-SCT modality could be widely accepted by each transplantation center as a standard approach for the treatment of hematological malignancies, IMD, and genetic diseases. More recently, our group demonstrated that the addition of low-dose PTCy to the Beijing Protocol can further enhance the G-CSF/ATG-induced GVHD protective activity, leading to a superior survival. A similar attempt has also been made by other researchers. In addition, with the deep understanding of the underlying mechanisms behind transplant complications, such as PGF, virus infection, and relapse, novel methods for the prevention and treatment of these complications will be established. All of these will further improve the outcomes of haplo-SCT. In this regard, well-designed prospective clinical trials are needed to compare the outcomes of the currently available haplo-SCT protocols and complication prevention and treatment methods, as well as to establish better treatments and prophylaxis for patients who had PGF, virus infection, and relapse after haploidentical allografts.

## Author Contributions

X-JH designed the study. X-YZ and Y-JC collected data, analyzed the data, and drafted the manuscript. All authors contributed to data interpretation, manuscript preparation, and approval of the final version.

### Conflict of Interest

The authors declare that the research was conducted in the absence of any commercial or financial relationships that could be construed as a potential conflict of interest.
